# Effects of uric acid-lowering therapy in patients with chronic kidney disease: A meta-analysis

**DOI:** 10.1371/journal.pone.0187550

**Published:** 2017-11-02

**Authors:** Xiaole Su, Boyang Xu, Bingjuan Yan, Xi Qiao, Lihua Wang

**Affiliations:** Renal Division, Shanxi Medical University Second Hospital, Taiyuan, Shanxi, China; Universidade de Sao Paulo Faculdade de Medicina, BRAZIL

## Abstract

**Background and objectives:**

The effects of uric acid-lowering therapy in patients with chronic kidney disease (CKD) remain uncertain. Therefore, we undertook a systematic review and meta-analysis to investigate the effects of uric acid-lowering agents on major clinical outcomes of CKD.

**Design, setting, participants, and measurements:**

According to the pre-specified protocol that was registered with PROSPERO (No. CRD42016038030), we searched systematically in MEDLINE, EMBASE, and the Cochrane Library for trials up to February 2016. Prospective, randomized, controlled trials assessing the effects of uric acid-lowering agents on cardiovascular and kidney outcomes in patients with CKD were included. Random-effects analytical methods were used.

**Results:**

Sixteen eligible trials were identified, providing data for 1,211 patients with CKD, including 146 kidney failure events and 69 cardiovascular events. Uric acid-lowering therapy produced a 55% relative risk (RR) reduction (95% confidence interval [95% CI], 31–64) for kidney failure events (*P* < 0.001), and a 60% RR reduction (95% CI, 17–62) for cardiovascular events (*P* < 0.001), but had no significant effect on the risk of all-cause death (RR, 0.86; 95% CI, 0.50–1.46). The mean differences in rate of decline in the estimated glomerular filtration rate (4.10 mL/min/1.73 m^2^ per year slower in uric acid-lowering therapy recipients, 95% CI, 1.86–6.35) and the standardized mean differences in the change in proteinuria or albuminuria (−0.23 units of standard deviation greater in uric acid-lowering therapy recipients; 95% CI, −0.43 to −0.04) were also statistically significant.

**Conclusions:**

Uric acid-lowering therapy seemed to improve kidney outcomes and reduce the risk of cardiovascular events in adults with CKD.

## Introduction

Chronic kidney disease (CKD) is a severe public health challenge. The unfavorable impact of CKD includes not only progression to end-stage renal disease (ESRD), but also increased risk of all-cause mortality and cardiovascular disease [1–3]. Management of the progression of CKD aims to address a multiplicity of factors that are involved [4]. Published data implicate elevated serum uric acid concentration with the evolution of CKD, or vice versa [5–7], and the relationship could be circular, with each worsening the other [8]. Meanwhile, hyperuricemia has been implicated as a cause of hypertension and insulin resistance [9, 10], which may also lead to kidney disease.

Whether elevated serum uric acid levels could be a consequence of CKD, a cause, or a marker of other risk factors that lead to kidney disease, is still under discussion. Uric acid-lowering therapy has been reported to delay the progression of kidney disease and/or reduce cardiovascular risk in patients with CKD [11–13] in several randomized controlled trials (RCTs). Conversely, no benefit from uric acid-lowering agents were concluded in a few other RCTs [14, 15]. Current RCTs are all suggestive smaller studies and several ongoing RCTs [16, 17] with a larger sample size and longer duration are expected to deliver their results in the near future. Two published meta-analyses have provided evidence of the relation between uric acid-lowering therapy and changes in the estimated glomerular filtration rate (eGFR) in populations with CKD; however, the results were discordant [18, 19]. Furthermore, both overviews did not evaluate the effects of uric acid-lowering therapy on dichotomous outcomes, including clinical kidney failure and cardiovascular events.

Thus, the issue of whether uric acid-lowering agents should be used in asymptomatic individuals with CKD for the specific purpose of delaying CKD progression or reducing cardiovascular events remains inconclusive and controversial, and it is difficult for clinicians to interpret and apply these results. In this systematic review, we sought to synthesize all the available data from clinical trials and evaluate the effects of uric acid-lowering agents on renal and cardiovascular outcomes in patients with CKD.

## Materials and methods

### Data sources and search strategy

This systematic review was performed according to a pre-specified protocol [20] registered at the International Prospective Register of Systematic Reviews (CRD42016038030) and the reporting was in line with Preferred Reporting Items for Systematic Reviews and Meta-Analyses (PRISMA) guidelines [21] (S1 Checklist). Relevant RCTs were identified and searched in major electronic database without language restriction: MEDLINE via Ovid (from 1946 to Feb 2016), EMBASE (from 1966 to Feb 2016), and the Cochrane Central Register of Controlled Trials (no date restriction). We used Medical Subject Headings (MeSH) and the keywords CKD, RCTs, and all spellings of uric acid-lowering drugs, including allopurinol, benzbromarone, probenecid, sulfinpyrazone, febuxostat, rasburicase, and pegloticase. Although some other agents, such as angiotensin receptor blockers [22] and statins [23, 24], may also reduce the levels of uric acid, the primary effects of these agents would influence the kidney and cardiovascular outcomes. Accordingly, relevant studies on these agents were excluded. A search on ClinicalTrials.gov was also used to identify the ongoing, but unpublished, studies in this field. We combined this strategy with a manual search of reference lists from identified trials and review articles.

### Study selection and outcome estimation

We included data from RCTs in which any uric acid-lowering agent was given to patients with CKD. These data were extracted from studies performed solely in people with CKD, as well as studies in which data on the CKD population could be obtained. We excluded those trials reporting a follow-up shorter than 6 months because we wanted to focus on longer-term outcomes.

The primary outcomes were kidney failure events, which were defined as a more than 25% or 50% decrease in the eGFR [25], and doubling of serum creatinine and ESRD during the follow-up period. The secondary outcomes included the following: 1. Rate of change in eGFR per year. The difference from baseline in eGFR divided by the number of years between creatinine measurements (mL/min/1.73m^2^ per year); 2. Changes in proteinuria or albuminuria from baseline to the end of follow-up, including urinary protein excretion, urinary albumin excretion, protein to creatinine, and albumin to creatinine ratios; 3. Cardiovascular events, including cardiovascular mortality, myocardial infarction, unstable angina, acute coronary syndromes, stroke, coronary revascularization procedures, peripheral revascularization procedures, heart failure requiring hospitalization, and stent thrombosis or comparable definitions used by the authors of each trial; 4. All-cause mortality; 5. Drug-related adverse events.

### Data extraction and quality assessment

Study selection, data extraction, and quality assessment were performed independently by two investigators (X.S. and B.X.), according to the prespecified study protocol [20]. The two investigators screened the titles and abstracts of records identified by the search strategies for eligibility. Disagreements were resolved by discussion with a third reviewer (L.W.). Data of the prespecified variables from the included studies were extracted into a computerized spreadsheet, including baseline study characteristics (design, follow-up duration, method of randomization, and withdrawals/dropouts); baseline patient characteristics (age, sex, CKD stage, mean proteinuria or albuminuria, eGFR, serum uric acid and creatinine concentrations); type, dose, and frequency of uric acid-lowering drugs used; outcome events; and adverse events.

We assessed sources of bias using the Cochrane Collaboration risk-of-bias tool [26, 27], including an assessment of financial conflicts of interest [28]. We developed operational definitions for high, low, and unclear risk of bias for each of the eight validity domains (S1 Text). Furthermore, the study quality was also quantified using the Jadad scale [29].

### Data synthesis and analysis

If individual study relative risks (RRs) were unavailable in the original article, RRs and 95% confidence intervals (CIs) for binary outcomes were calculated from event numbers extracted from each trial before data pooling. In calculating the RR values, we used the total number of patients randomized in each group as the corresponding denominator. We pooled risk estimates from individual trials using the Der Simonian-Laird random effects model [30]. Considering that the Der Simonian-Laird procedure can be unstable with small numbers of studies [31], a restricted maximum likelihood [32] or the empirical Bayes procedure [33] (equivalent to iterated Der Simonian-Laird) were applied to analyze the summary effects as a part of the sensitivity analysis. Where continuous scales of measurement were used, the mean differences with 95% CI were used to pool eGFRs, and the standardized mean differences with 95% CI were used to pool the proteinuria or albuminuria data.

We carried out the following prespecified sensitivity analyses [20], using different random-effects estimation methods as above mentioned: exclusion of trials with sample sizes less than 50; exclusion of trials with follow-up years less than 12 months; and exclusion of trials with Jadad scores less than 2. Heterogeneity across the included trials was analyzed using *I*-squared or *τ*-squared statistics. We explored potential heterogeneity using prespecified subgroup analyses [20], including mean age, follow-up time, different uric acid-lowering agents, and baseline mean serum urate. Other analyses were *post-hoc*: number of participants and differences in the mean changes in serum urate. Between-group heterogeneity was assessed by the Chi-square test and metaregression [34]. A two-sided *P*-value < 0.05 was considered statistically significant. Stata version 14.0 was used for all statistical analyses (StataCorp LP, College Station, TX, USA).

## Results

### Search results and characteristics of included studies

**Fig 1:**
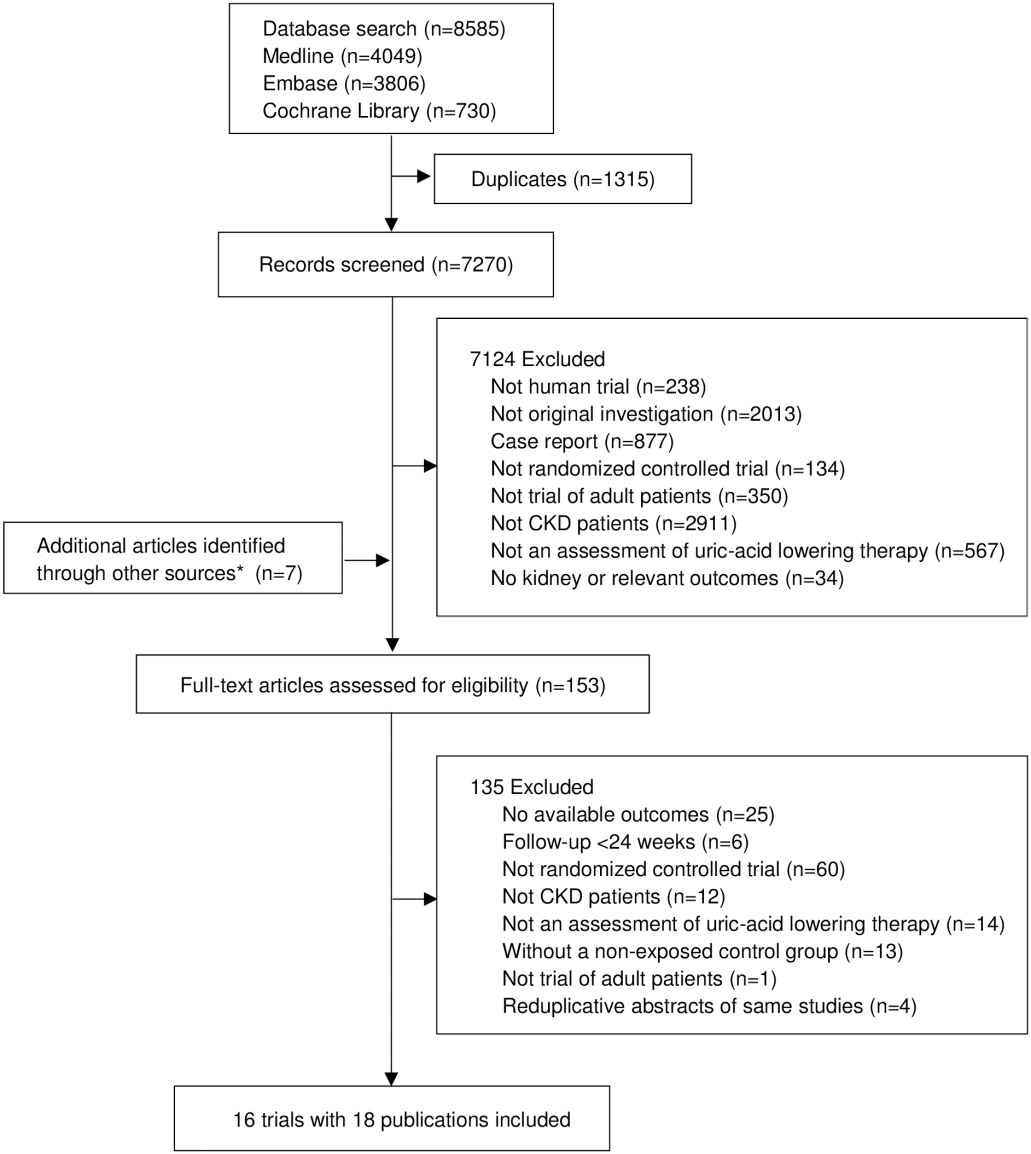
Flow diagram of the search selection. CKD, chronic kidney disease. *Identified from references of other review articles.

The primary electronic search strategy found 8 585 records. Once duplicates had been removed, 7 270 abstracts were screened, and 153 publications were selected for full-text review, including seven trials identified from three review articles [18, 19, 35]. This process yielded 18 publications [11–15, 36–48] from 16 RCTs with 1 211 CKD patients (Fig 1). Table 1 summarizes the characteristics of the included studies. Median follow-up duration was 12 months (range, 6–84 months). Of the 16 RCTs, three different uric acid-lowering agents were studied: allopurinol, febuxostat, and pegloticase. Twelve trials [11, 13–15, 36, 40–43, 45, 46, 48] studied allopurinol, one trial [12] studied febuxostat, one trial [47] studied pegloticase, and the others [38, 39] compared allopurinol with febuxostat. The decline in serum urate ranged from 0.93 to 4.23 mg/dL.

**Table 1:** Characteristics of included trials and patient.

Study (year)	Inclusion criteria	Outcome	Size of study	Treatment group (mg/d)	Control group	Mean follow-up months	Men (%)	Mean age (years)	Definition of CKD	Baseline mean SUA (mg/dL)	Baseline Scr (mg/dL)	Difference of SUA decline (mg/dL)^b^	Jadad scores	Funding source
Siu YP, (2006) [13]	UPE>0.5g/d and/or Scr >1.35mg/dL	ESRD, all-cause mortality, change of UPE, change of sUA	51	Allopurinol, 100–200mg/d	Standard care	12	43	48	Not defined	9.84	1.75	4.03	3	Non-industry
Liu J, (2007) [41]	Scr 120–400μmol/L, sUA: men>420μmol/L, women>350μmol/L	ESRD, all-cause mortality, change of UPE, change of sUA	47	Allopurinol, 100–200mg/d	Standard care	12	62	46	Not defined	9.83	1.71	4.01	2	NA
Sarris E, (2007) [42]	sUA>7mg/dL, Scr 1.5–3.0mg/dL	all-cause mortality, change of sUA	36	Allopurinol, 150mg/d	Free of treatment	12	47	50	Not defined	9.02	1.89	4.23	1	NA
Lei J, (2009) [40]	Scr 133–442μmol/L, sUA: men>420μmol/L, women>360μmol/L	ESRD & Scr double, all-cause mortality, change of sUA	57	Allopurinol, 100–200mg/d	Standard care	12	68	49	Not defined	8.78	2.71	2.43	2	NA
Zhou DY, (2009) [48]	sUA: men>417μmol/L, women>357μmol/L, UPE>0.5g/d or eGFR<60ml/min	rate of change in eGFR, change of sUA, change of UPE	86	Allopurinol, ≤200mg/d	Standard care	6	43	59	Not defined	7.05	1.34	1.13	2	NA
Deng YH, (2010) [36]	Scr 133–442μmol/L, sUA: men 420–600μmol/L, women 360–600μmol/L	ESRD & Scr double, all-cause mortality, change of sUA	61	Allopurinol, 100–300mg/d	Standard care	12	53	59	Not defined	8.78	2.52	2.75	2	NA
Shen H, (2010) [43]	Scr 133–442μmol/L, sUA: men>420μmol/L, women>350μmol/L	ESRD, all-cause mortality, change of sUA	51	Allopurinol, 100–200mg/d	Standard care	12	67	47	Not defined	8.95	2.68	2.43	1	NA
Kao MP, (2011) [14]	LVMI: men≥115g/m^2^, women≥95g/m^2^, eGFR 30–60ml/min	ESRD or kidney failure events, all-cause mortality, rate of change in eGFR, change of sUA, change of PCR	53	Allopurinol, 300mg/d	Placebo	9	53	72	Not defined	7.23	44.98^a^	3.37	3	Non-industry
Shi YJ, (2011) [15]	age:18–70, biopsy-proven IgAN, UPE 0.15–2.0g/d, serum Alb>3.5g/dL, Scr<3mg/dL, BP<180/100mmHg, sUA men>7mg/dL, women>6mg/dL	ESRD, Scr double, all-cause mortality, rate of change in eGFR, change of sUA, change of PCR	40	Allopurinol, 100–300mg/d	Placebo	6	55	40	IgAN	7.85	1.35	1.80	3	Non-industry
Tan Y, (2011) [45]	DM, age>18, eGFR 30–60ml/min, UPE>0.5g/d,sUA: men 420–600μmol/L, women 360–600μmol/L	ESRD & Scr double, all-cause mortality, change of sUA	140	Allopurinol, (No exact dose)	Standard care	24	51	59	DKD	8.77	2.53	3.41	2	NA
Goldfarb DS, (2013) [38]	uUA>700mg/d, age>18, history of kidney stones, calcium kidney stone ≥3 mm in its longest	all-cause mortality, change of sUA, change of UPE	99	Allopurinol, 300mg/d; or Febuxostat, 80mg/d	Placebo	6	86	47	Kidney stone	6.27	1.01	2.24	3	Industry
Ivanov DD, (2013) [39]	asymptomatic hyperuricemia, CKD2–3	rate of change in eGFR, change of sUA, change of ACR	56	Allopurinol, 300mg/d;or Febuxostat, 80mg/d	Free of treatment	14	NA	NA	Not defined	NA	NA	1.19	1	NA
Tuta L, (2014) [46]	eGFR 30–59ml/min	CV events, rate of change in eGFR	125	Allopurinol, 100mg/d	Standard care	12	NA	NA	Not defined	NA	NA	NA	1	NA
Yood RA, (2014) [47]	age≥18, sUA≥8.0mg/dL, CKD stage 3–4	rate of change in eGFR	103	Pegloticase 8mg/2w, or 8mg/4w	Placebo	6	71	62	Not defined	NA	40.84^a^	NA	5	Industry
Goicoechea, (2015) [11]	eGFR<60ml/min	ESRD & 50% decline in eGFR, CV events, all-cause mortality, rate of change in eGFR, change of sUA, change of UAE	113	Allopurinol, 100mg/d	Standard care	84	NA	72	Not defined	7.55	1.75	1.90	3	No funding supported
Sircar D, (2015) [12]	age 18–65, eGFR 15–60ml/min, sUA≥7mg/dL	ESRD, CV events, all-cause mortality, rate of change in eGFR, change of sUA	93	Febuxostat, 40mg/d	Placebo	6	71	57	Not defined	8.59	2.11	3.40	5	No funding supported

ACR, albumin to creatinine ratio; Alb, albumin; BP, blood pressure; CKD, chronic kidney disease; CV, cardiovascular; DKD, diabetic kidney disease; DM, diabetes mellitus; eGFR, estimated glomerular filtration rate; ESRD, end stage renal disease; IgAN, IgA nephropathy; LVMI, left ventricular mass index; NA, not available; PCR, protein to creatinine ratio; Scr, serum creatinine; UA, serum uric acid; UPE, urinary protein excretion; uUA, urinary uric acid.

^a^Only eGFR available;

^b^Between treatment and control groups.

### Risk of bias of the included studies

The methodological quality of the included trials was not high overall and varied substantially. Seven trials [11–15, 38, 47] had a Jadad scale of 3 to 5; the others scored less than 3 (Table 1). The results from the Cochrane Collaboration risk-of-bias tool are shown in S1 and S2 Figs. Overall, the proportion of trials with low risk of bias was 31% in terms of random sequence generation, 13% in terms of allocation concealment, 25% in terms of blinding of both participants and health care professionals, 13% in terms of blinding of outcome assessors, 81% in terms of attrition, and 75% in terms of reporting bias. With respect to conflicts of interest, 13% of the RCTs were funded by the pharmaceutical industry and 13% reported author-industry financial relationships. To investigate reporting/publication bias, we searched and found 41 protocols for 153 full-text reviewed articles. In 25 studies without reporting the available outcome, we did not find a preplanned available outcome.

### Effects of uric acid-lowering therapy on kidney outcomes

**Fig 2:**
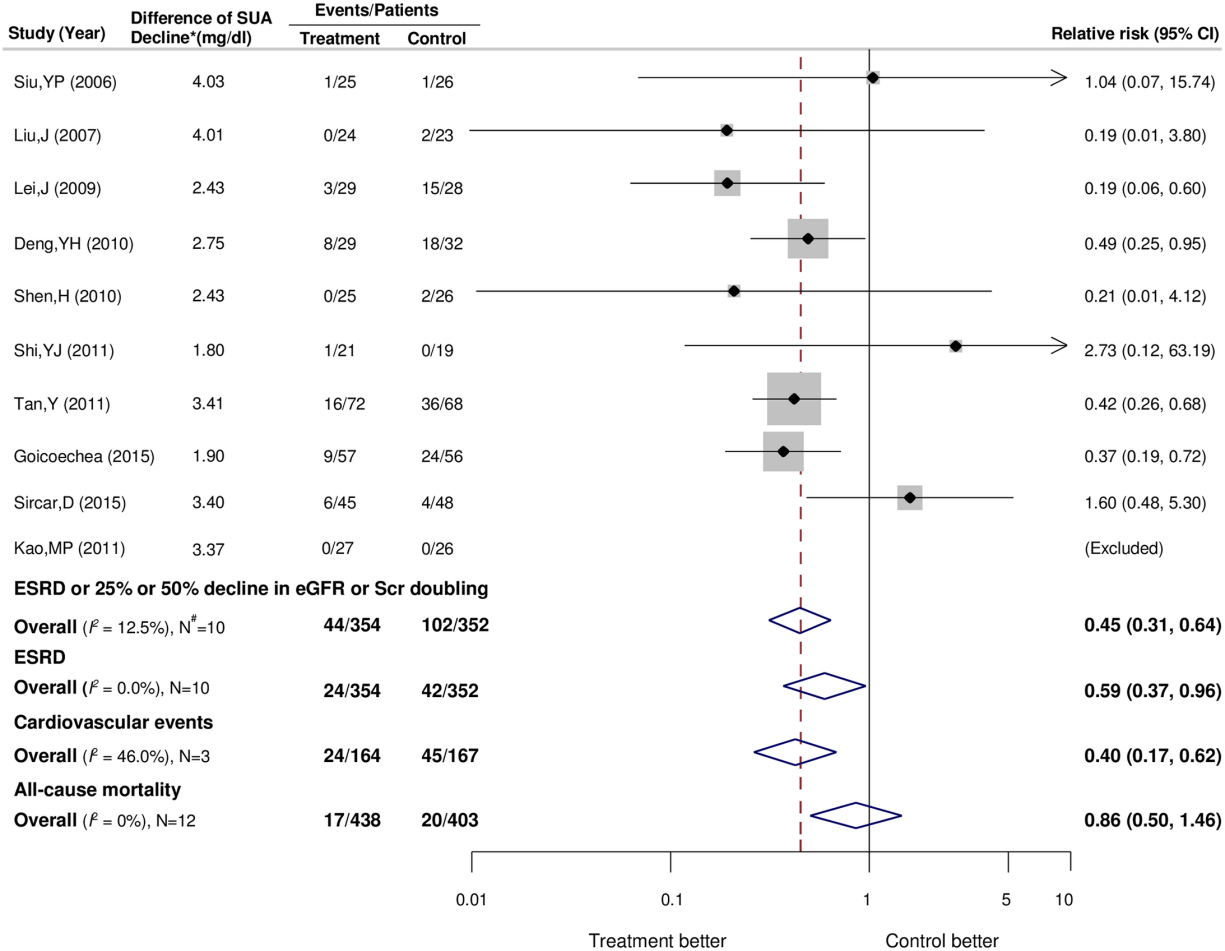
Forest plot for the kidney failure events, cardiovascular events and all-cause mortality. CI, confidence interval; eGFR, estimated glomerular filtration rate; ESRD, end-stage renal disease; Scr, serum creatinine; SUA, serum uric acid. *Between treatment and control groups. #Number of trials.

Kidney failure events were reported in 10 trials [11–15, 36, 40, 41, 43, 45], including 706 participants, among whom 146 events were observed. As shown in Fig 2, uric acid-lowering therapy reduced the risk of kidney failure events by 55% (RR, 0.45; 95% CI, 0.31–0.64) compared with standard treatment or placebo, without evidence of heterogeneity (*I*^*2*^ = 12.5%; *P* for heterogeneity = 0.33). As a component of kidney failure events, ESRD events were reported in 10 trials [11–15, 36, 40, 41, 43, 45], including 706 patients and 66 events. Uric acid-lowering therapy reduced the risk of ESRD by 41% (RR, 0.59; 95% CI, 0.37–0.96; Fig 2) compared with standard treatment or placebo, with no significant heterogeneity (*I*^*2*^ = 0%).

**Fig 3:**
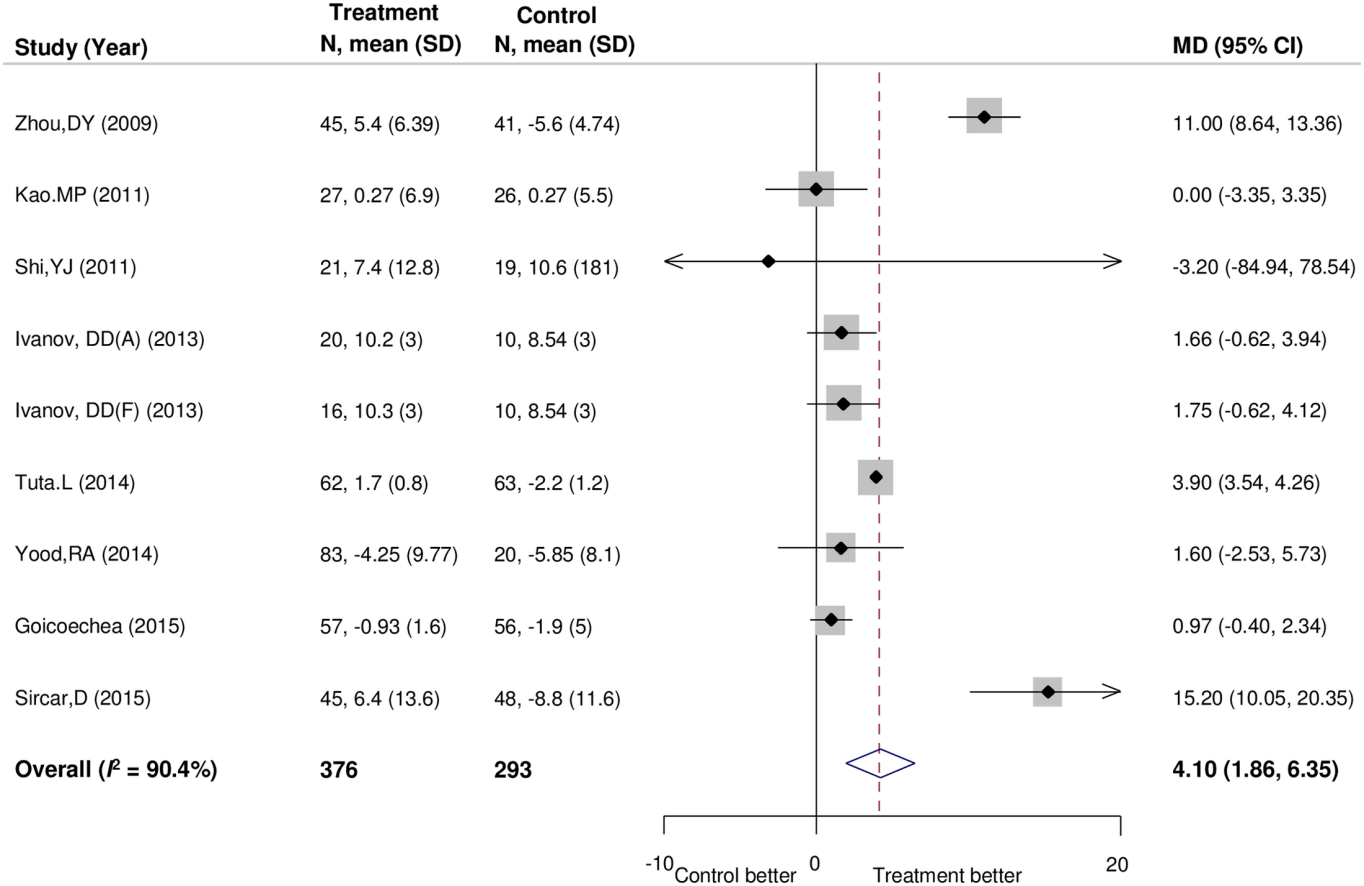
Forest plot for the rate of change in estimated glomerular filtration rate (eGFR). Positive values in difference of change represent slower decline for eGFR in uric acid-lowering therapy group than in control group. Ivanov, DD(A) and Ivanov, DD(F) were subgroups of Ivanov, DD (2013) study. CI, confidence interval; MD, mean difference; SD, standard deviation.

Eight trials [11, 12, 14, 15, 39, 46–48] involving 669 participants reported the effects of uric acid-lowering therapy on the rate of change in eGFR, one of which was analyzed as the second outcome [14]. When compared with the control group, uric acid-lowering agents showed a significant effect on slowing the rate of eGFR decline by 4.10 mL/min/1.73m^2^ per year (95% CI, 1.86–6.35; Fig 3). High amounts of heterogeneity were detected (*I*^*2*^ = 90.4%; *P* for heterogeneity < 0.001). Subgroup analyses did not show any differences between studies using the predefined characteristics (Table 2). The smaller sample size and fewer trials meant that prespecified subgroup analyses could be performed only partly.

**Table 2:** Subgroup analysis of kidney function by outcome.

Subgroup	No. of trials	n	WMD/SMD (95%CI)	Mean uric-acid difference, mg/dL	*P* for WMD/SMD	*I*^*2*^	*P* for heterogeneity test^a^
**Rate of Change in eGFR**							
**Different uric acid-lowering drugs**							
Allopurinol	6	447	3.54 (0.91, 6.18)	1.88	0.008	91.7%	0.56
Febuxostat	2	119	8.27 (–4.90, 21.45)	2.30	0.218	95.4%	
Pegloticase	1	103	1.60 (–2.53, 5.73)	NA	0.447	NA	
**Follow-up time**							
<12 months	5	375	6.79 (0.10, 13.49)	2.43	0.047	90.9%	0.67
≥12 months	4	294	2.19 (0.31, 4.07)	1.43	0.023	86.3%	
**Difference of SUA decline**^b^							
<1.8mg/dl	3	142	4.80 (–1.24, 10.84)	1.17	0.119	95.0%	0.87
≥1.8mg/dl	4	299	4.87 (–1.71, 11.46)	2.62	0.147	89.5%	
**Change of Proteinuria or Albuminuria**							
**Different uric acid-lowering drugs**							
Allopurinol	7	357	–0.17 (–0.39, 0.04)	2.54	0.114	0%	0.15
Febuxostat	2	75	–0.59 (–1.10, –0.07)	1.72	0.025	0%	
**Age**							
<48	4	186	–0.12 (–0.44, 0.19)	2.57	0.435	0%	0.58
≥48	3	190	–0.24 (–0.55, 0.08)	2.84	0.137	15.4%	
**Baseline SUA**							
<7.23 mg/dl	3	185	–0.41 (–0.73, –0.09)	1.87	0.011	0%	0.07
≥7.23 mg/dl	4	191	–0.01 (–0.30, 0.27)	3.30	0.934	0.0%	
**Follow-up time**							
<12 months	5	278	–0.32 (–0.57, –0.07)	2.16	0.011	0%	0.25
≥12 months	4	154	–0.14 (–0.58, 0.31)	2.61	0.545	43.7%	
**Difference of SUA decline**^b^							
<2.24mg/dl	4	182	–0.40 (–0.70, –0.10)	1.33	0.009	0%	0.15
≥2.24mg/dl	5	250	–0.11 (–0.37, 0.16)	3.18	0.430	0%	

Positive values in difference of the change represent slower decline in eGFR in uric acid-lowering therapy group than in control group. Negative values in difference of the change represent greater decreases for proteinuria or albuminuria in uric acid-lowering therapy group than in control group. Subgroups of age and baseline serum urate in rate of change in eGFR analysis were not analyzed due to insufficient data. CI, confidence interval; eGFR, estimated glomerular filtration rate; NA, not available; n, number of participants; SMD, standardized mean difference; SUA, serum uric acid; WMD, weighted mean difference.

^a^*P* value calculated by *χ*2 statistics was shown. Statistical significance of results from meta regression was consistent.

^b^Between treatment and control groups

**Fig 4:**
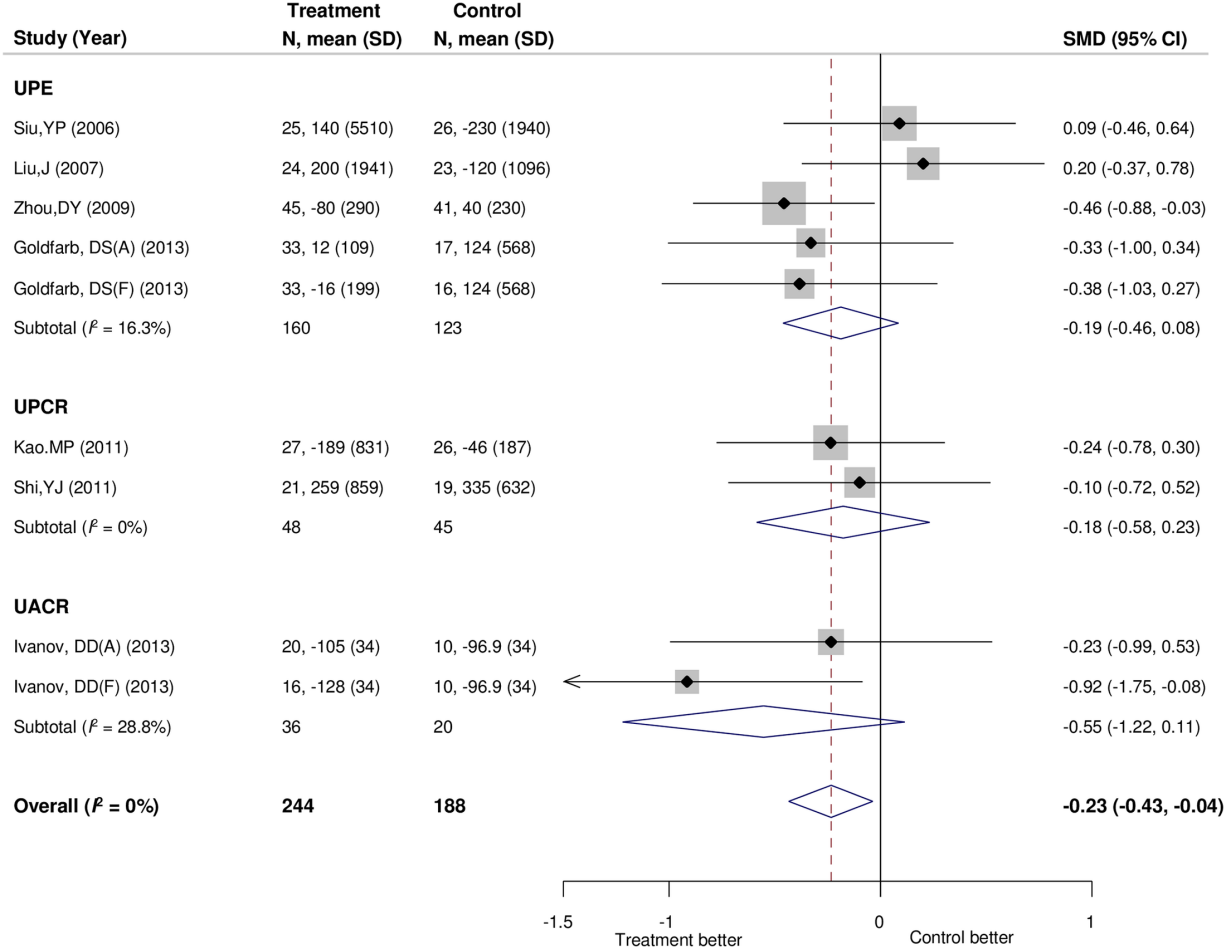
Forest plot for the change in proteinuria or albuminuria. Negative values in difference of change represent greater decreases for proteinuria or albuminuria in uric acid-lowering therapy group than in control group. Goldfarb, DS(A) and Goldfarb, DS(F) were subgroups of Goldfarb, DS (2013) study. Ivanov, DD(A) and Ivanov, DD(F) were subgroups of Ivanov, DD (2013) study. CI, confidence interval; SD, standard deviation; SMD, standard mean difference.

Seven trials [13–15, 38, 39, 41, 48] comprising 432 participants provided data for albuminuria or proteinuria. Among them, the data for urinary protein excretion was provided in four trials [13, 38, 41, 48] with 283 participants; the data for the protein to creatinine ratio in two trials [14, 15] with 93 participants; and the albumin to creatinine ratio in one trial [39] with 56 participants. The standardized mean difference in albuminuria or proteinuria was statistically significant (−0.23 units of standard deviation; 95% CI, −0.43 to −0.04; *I*^*2*^ = 0%; Fig 4).

### Effects of uric acid-lowering therapy on cardiovascular events and all-cause mortality

Sixty-nine cardiovascular events were reported in only three trials [11, 12, 46] with 331 participants. Overall, compared with placebo or usual-care control groups, uric acid-lowering therapy produced a 60% reduction in the risk of cardiovascular events (RR, 0.40; 95% CI, 0.17–0.62; Fig 2), without evidence of heterogeneity (*I*^*2*^ = 0%). Data were available for 37 events of all-cause death in 12 trials [11–15, 36, 38, 40–43, 45] (841 participants). Our analysis showed no clear effect of uric acid-lowering therapy on the risk of all-cause death (RR, 0.86; 95% CI, 0.50–1.46; *I*^*2*^ = 0%; Fig 2).

### Adverse effects

In all the included trials, no severe adverse events were reported. Mild to moderate transient adverse events related to uric acid-lowering therapy, including skin rash, arthralgia, gastrointestinal symptoms, and elevation of liver function enzyme, were reported in 12 trials [11–15, 36, 38, 41–43, 45, 48], at rates of 2.21, 1.67, 1.72, and 1.55%, respectively.

### Sensitivity analysis

As shown in Table 3, almost all the results did not vary substantially according to the prespecified characteristics in the sensitivity analysis, including omitting studies with follow-up less than 12 months, omitting studies with a sample size less than 50 participants, omitting studies with a Jadad score less than or equal to 1, and using different random-effects estimation methods. A notable exception was that the effects of uric acid-lowering therapy on albuminuria or proteinuria became statistically non-significant compared with the control group in some sensitivity analyses.

**Table 3:** Sensitivity analyses of kidney function by outcome.

	Kidney failure events				ESRD				eGFR				Proteinuria			
	n/No.	*τ*^2^	RR	95% CI	n/No.	*τ*^*2*^	RR	95% CI	n/No.	*τ*^*2*^	MD	95% CI	n/No.	*τ*^*2*^	SMD	95% CI
**Base-case**	706/10	0.04	0.45	0.31, 0.64	706/10	<0.001	0.59	0.37, 0.96	669/8	8.48	4.10	1.86, 6.35	432/7	<0.001	–0.23	–0.43, –0.04
**Omit sample size less than 50**	619/8	0.05	0.44	0.30, 0.65	619/8	<0.001	0.61	0.38, 0.99	629/7	8.59	4.11	1.85, 6.37	345/5	<0.001	–0.32	–0.54, –0.09
**Omit follow-up less than 12 months**	520/7	<0.001	0.40	0.29, 0.54	520/7	<0.001	0.49	0.29, 0.83	294/3	2.95	2.19	0.31, 4.07	154/3	0.09	–0.14	–0.58, 0.31
**Omit Jadad scores with 1**	655/9	0.06	0.46	0.31, 0.67	655/9	<0.001	0.61	0.38, 0.99	488/6	35.11	5.54	0.14, 10.95	376/6	<0.001	–0.19	–0.40, 0.02
**Different statistical methods**																
**DL**	706/10	0.04	0.45	0.31, 0.64	706/10	<0.001	0.59	0.37, 0.96	669/8	8.48	4.10	1.86, 6.35	432/7	<0.001	–0.23	–0.43, –0.04
**REML**	653/9	<0.001	0.44	0.32, 0.60	613/8	<0.001	0.59	0.37, 0.96	669/8	23.57	4.30	0.78, 7.81	432/7	<0.001	–0.23	–0.43, –0.04
**EB**	653/9	0.06	0.45	0.31, 0.66	613/8	<0.001	0.59	0.37, 0.96	669/8	22.49	4.29	0.85, 7.73	432/7	<0.001	–0.23	–0.43, –0.04

Kidney failure events was defined as doubling of serum creatinine level or 50% decline in estimated GFR or end-stage renal disease. GFRs expressed in mL/min/1.73 m^2^. Sensitivity analyses of cardiovascular events and all-cause mortality were not performed due to smaller sample size and fewer trials. CI, confidence intervals; DL, DerSimonian-Laird; EB, empirical Bayes; eGFR, estimated glomerular filtration rate; ESRD, end stage renal disease; MD, mean difference; n, number of patients; No. number of trials; REML, restricted maximum likelihood; RR, relative risk; SMD, standardized mean difference.

## Discussion

Hyperuricemia is common in patients with CKD, and whether uric acid-lowering therapy is required by patients with CKD to delay progression of CKD or decrease adverse cardiovascular outcomes has not been established completely over recent years. In this meta-analysis of 16 trials involving 1 211 patients with CKD, treatment with uric acid-lowering agents compared with placebo or usual-care resulted in a 55% relative reduction in the risk of kidney failure events, a 60% reduction in cardiovascular events, a mild reduction in proteinuria (0.23 units of standard deviation), and rate of decline in eGFR of 4.10 ml/min/1.73m^2^ per year. These significant benefits were consistent across major subgroups and sensitivity analyses. No significant effect was observed on the risk of all-cause mortality. Mild, transient, and uncommon adverse effects would not affect these benefits in the treatment in patients with CKD. This finding was surprising given that the risks of kidney failure and cardiovascular events were reduced by more than half. However, we assumed that these results should be interpreted with caution, because the included studies were limited as follows: low or very-low quality of contributing studies (9/16 trials with a Jadad score less than 3); a considerable proportion of missing baseline patient characteristics; heterogeneity in baseline kidney function, cause of CKD, duration of follow-up (6–84 months) and the definitions and assessments of kidney and cardiovascular outcomes across these studies; small sample size (all less than 200) and a low number of events, especially in cardiovascular outcomes, comprising only three trials with 69 events. The suboptimal quality of the included trials limited our ability to reach robust conclusions.

Although it was possible that the positive effects of uric acid-lowering therapy on populations with CKD found in this study were limited, a similar association between kidney function and the benefit of uric acid-lowering therapy has been described in several studies [49, 50], which suggested a potentially important clinical relation. In a *post-hoc* analysis of the Reduction of Endpoints in Non-Insulin-Dependent Diabetes Mellitus With the Angiotensin II Antagonist Losartan (RENAAL) Study (1342 participants with diabetic nephropathy; median follow-up, 3.4 years) [22], the researchers found that each 0.5 mg/dL reduction in serum uric acid concentration was associated with a 6% (95% CI: 3–10) reduction in the risk of either doubling serum creatinine or onset of ESRD. Goicoechea et al. [11] performed a 7-year randomized study in 113 individuals with eGFR < 60 mL/min/1.73 m^2^. Allopurinol was shown to reduce the risk of kidney failure events (initiating dialysis therapy, and/or doubling serum creatinine level, and/or 50% decrease in eGFR) by 68% and the risk of cardiovascular events by 57%, which agrees with our study. In a recent meta-analysis of 19 RCTs enrolling 992 patients with CKD stage 3–5 [19], allopurinol reduced serum uric acid levels and blood pressure significantly, with a more favorable eGFR, compared with the controls. In another meta-analysis of eight RCTs [18], however, allopurinol had no effect on eGFR compared with the controls in five trials (n = 346) but abrogated increases in serum creatinine in three trials (n = 130). Notably, the literature searches did not capture trials published in languages other than English.

Another possible beneficial effect from uric acid-lowering therapy is the reduction of cardiovascular risk. Numerous epidemiological studies have investigated the link between hyperuricemia and the incidence of major cardiovascular endpoints [6, 9, 51]; however, the relationship in the CKD population is conflicting because of the complicated interactions between serum uric acid levels and kidney function. An analysis published in 1999 of data from 6 763 subjects in the Framingham Heart Study cohort [52] observed a lack of association between uric acid and cardiovascular endpoints, which was likely because of major confounding factors, such as decreased GFR. In the First National Health and Nutrition Examination Survey (NHANES 1) [51], this association between uric acid levels and cardiovascular mortality was attenuated from 1.48 (95% CI: 1.13–1.96) to 1.25 (0.89–1.75), with hazard ratio and the statistical significance disappearing after adjustment for the albumin-creatinine ratio and eGFR. There were insufficient data on cardiovascular events (only three trials with 69 events) for the meta-analysis, although the 60% reduction cardiovascular events was explored. Only mild to moderate adverse effects of study drugs in our study might be related to the small sample size and short follow-up duration. However, adverse effects must always be kept in focus while prescribing this drug, particularly for allopurinol. Caution is needed in interpreting these results.

To the best of our knowledge, the current study represents the largest systematic review of uric acid-lowering treatment administration on kidney and cardiovascular outcomes, and is the first meta-analysis to evaluate the effects of uric acid-lowering treatments on kidney failure events and cardiovascular risk in a population with CKD. Our study may provide some additional information with clinical evidence and form the basis for future research. It appears that decreasing serum uric acid levels is an alternative intervention to delay the progression of CKD and reduce cardiovascular events. Thus, lowering uric acid might represent a new therapeutic avenue in the population with CKD. Larger and well-designed RCTs are needed to confirm these benefits and to establish formally the effects of serum uric acid lowering on hard cardiovascular and renal endpoints.

There are several limitations to our study. First, as has been noted, the included studies were of low quality, such as the lack of placebo control groups (only 5/16 trials). Different studies included the specific participants, such as IgA nephropathy [15] and diabetic nephropathy [45], and it’s not possible to use these data to all patients with chronic kidney disease. We need to acknowledge the low-quality trials and clinical heterogeneity limited the application and generalization of the conclusions. Prospective randomized trials should focus on relatively homogeneous patient populations, such as those with diabetes mellitus, or whether patients with different stages of CKD would benefit similarly or differently from uric acid-lowering therapy. Second, most of trials (15/16) were associated with xanthine oxidase inhibitors, including allopurinol and febuxostat. As discussed, it is possible that the favorable results for allopurinol and febuxostat might be related to an inhibitory effect on reactive oxygen species rather than lowering serum uric acid levels. The renoprotective effects from the inhibition of reactive oxygen species formation by xanthine oxidase inhibitors have not been discounted [53]. Among other uric acid-lowering agents, such as benzbromarone, lesinurad [54], and canagliflozin (sodium glucose transporter 2) [55], whether there is heterogeneity in terms of their effects on kidney and cardiovascular protection in patients with CKD requires confirmation with further studies. Third, the absence of patient-specific data and the varying design of the included studies are limitations of many meta-analyses; in an effort to nullify these factors, we included only RCTs.

In conclusion, our meta-analysis indicated that uric acid-lowering therapy might improve kidney outcomes, including kidney failure events, proteinuria, and the rate of change in eGFR, and seem to reduce the risk of cardiovascular events in adults with CKD. The limitations of included studies (low quality and considerable clinical heterogeneity) meant that the results are not conclusive. Larger and well-designed RCTs of uric acid-lowering therapy are warranted to assess the precise renoprotective and cardiovascular protective effects of the therapy in a population with CKD.

## Supporting information

S1 Checklist PRISMA 2009 checklist. (DOC)

S1 Text Risk of bias for the outcome. (DOCX)

S2 Text Electronic search strategy. (DOCX)

S1 Fig Risk of bias graph. Only abstract was available in Sarris.E 2007, Ivanov, DD 2013 and Tuta, L 2014.
(PNG)

S2 Fig Risk of bias summary. (PNG)
