# Supplementary material for: Effects of uric acid-lowering therapy in patients with chronic kidney disease: A meta-analysis
Source: PLoS One. 2017 Nov 2;12(11):e0187550. doi: 10.1371/journal.pone.0187550 (PMC5667873; doi:10.1371/journal.pone.0187550)
Supplement: S2 Text — (DOCX) [file pone.0187550.s003.docx]

**S2 Text: Electronic Search Strategy**

**Ovid Medline (1946 to Feb Week2 2016)**

60 29 and 44 and 59

59 54 not 58

58 56 not 57

57 55 and 56

56 Animals/

55 Humans/

54 46 or 47 or 48 or 49 or 50 or 51 or 52 or 53

53 groups.ab.

52 trial.ab.

51 randomly.ab.

50 drug therapy.fs.

49 placebo.ab.

48 randomized.ab.

47 controlled clinical trial.pt.

46 randomized controlled trial.pt.

45 29 and 44

44 30 or 31 or 32 or 33 or 34 or 35 or 36 or 37 or 38 or 39 or 40 or 41 or 42 or 43

43 pegloticase$.mp.

42 peg-uricase$.mp.

41 rasburicase$.mp.

40 uricase$.mp.

39 febuxostat$.mp.

38 sulfinpyrazone$.mp.

37 probenecid$.mp.

36 benzbromarone$.mp.

35 ("xanthine oxidase inhibitor" or "xanthine oxidase inhibitors").mp.

34 oxypurinol$.mp.

33 allopurinol$.mp.

32 ("uric acid" adj6 lowering).mp.

31 exp Urate Oxidase/

30 gout suppressants.mp. or exp Gout Suppressants/

29 1 or 2 or 3 or 4 or 5 or 6 or 7 or 8 or 9 or 10 or 11 or 12 or 13 or 14 or 15 or 16 or 17 or 18 or 19 or 20 or 21 or 22 or 23 or 24 or 25 or 26 or 27 or 28

28 ((kidney or renal) adj (transplant* or graft* or allograft*)).mp.

27 exp Kidney Transplantation/

26 uremia.mp. or exp Uremia/

25 ("pre dialysis" or pre-dialysis).mp.

24 (CAPD or CCPD or APD).mp.

23 (CKF or CKD or CRF or CRD).mp.

22 (ESRF or ESKF or ESRD or ESKD).mp.

21 peritoneal dialysis.mp.

20 (hemodiafiltration or haemodiafiltration).mp.

19 (hemofiltration or haemofiltration).mp.

18 (haemodialysis or hemodialysis).mp.

17 exp Renal Dialysis/

16 exp Renal Insufficiency/

15 exp Renal Replacement Therapy/

14 albuminuria$.mp.

13 proteinuria$.mp.

12 creatinine$.mp.

11 (hyperuricaemia or hyperuricemia).mp.

10 "uric acid".mp.

9 (scr or cr or ccr).mp.

8 glomerular filtration rate.mp. or exp Glomerular Filtration Rate/

7 $gfr.mp.

6 exp Kidney Function Tests/

5 ((kidney or renal) adj (disease* or failure)).mp.

4 exp Kidney Diseases/

3 renal.mp.

2 kidney.mp.

1 exp Kidney/

**EMBASE (1966 to Feb 2016)**

45 43 and 44

44 (random* or blind* or placebo or "meta analysis").mp.

43 27 and 42

42 28 or 29 or 30 or 31 or 32 or 33 or 34 or 35 or 36 or 37 or 38 or 39 or 40 or 41

41 pegloticase$.mp.

40 peg-uricase$.mp.

39 rasburicase$.mp.

38 uricase$.mp.

37 febuxostat$.mp.

36 sulfinpyrazone$.mp.

35 probenecid$.mp.

34 benzbromarone$.mp.

33 ("xanthine oxidase inhibitor" or "xanthine oxidase inhibitors").mp.

32 oxypurinol$.mp.

31 allopurinol$.mp.

30 ("uric acid" adj6 lowering).mp.

29 gout suppressants.mp. or exp antigout agent/

28 exp urate oxidase/

27 1 or 2 or 3 or 4 or 5 or 6 or 7 or 8 or 9 or 10 or 11 or 12 or 13 or 14 or 15 or 16 or 17 or 18 or 19 or 20 or 21 or 22 or 23 or 24 or 25 or 26

26 ((kidney or renal) adj6 (transplant* or graft* or allograft*)).mp.

25 exp kidney transplantation/

24 uremia.mp. or exp uremia/

23 ("pre dialysis" or pre-dialysis).mp.

22 (CAPD or CCPD or APD).mp.

21 (CKF or CKD or CRF or CRD).mp.

20 (ESRF or ESKF or ESRD or ESKD).mp.

19 peritoneal dialysis.mp.

18 (hemodiafiltration or haemodiafiltration).mp.

17 (hemofiltration or haemofiltration).mp.

16 (hemodialysis or haemodialysis).mp.

15 exp kidney failure/

14 exp renal replacement therapy/

13 albuminuria$.mp.

12 proteinuria$.mp.

11 creatinine$.mp.

10 (hyperuricaemia or hyperuricemia).mp.

9 "uric acid".mp.

8 (scr or cr or ccr).mp.

7 glomerular filtration rate.mp. or exp glomerulus filtration rate/

6 $gfr.mp.

5 exp kidney function test/

4 ((kidney or renal) adj (disease* or failure)).mp.

3 exp kidney disease/

2 renal.mp.

1 kidney.mp. or exp kidney/

**CENTRAL**

#1 MeSH descriptor: [Kidney] explode all trees

#2 kidney:ti,ab,kw in Trials

#3 renal:ti,ab,kw

#4 MeSH descriptor: [Kidney Diseases] explode all trees

#5 (kidney or renal) next (disease* or failure):ti,ab,kw

#6 MeSH descriptor: [Kidney Function Tests] explode all trees

#7 *gfr:ti,ab,kw

#8 MeSH descriptor: [Glomerular Filtration Rate] explode all trees

#9 "glomerular filtration rate":ti,ab,kw

#10 scr or ccr or cr:ti,ab,kw

#11 "uric acid":ti,ab,kw

#12 hyperuricaemia or hyperuricemia:ti,ab,kw

#13 creatinine*:ti,ab,kw

#14 proteinuria*:ti,ab,kw

#15 albuminuria*:ti,ab,kw

#16 MeSH descriptor: [Renal Replacement Therapy] explode all trees

#17 MeSH descriptor: [Renal Insufficiency] explode all trees

#18 MeSH descriptor: [Renal Dialysis] explode all trees

#19 hemodialysis or haemodialysis:ti,ab,kw

#20 hemofiltration or haemofiltration:ti,ab,kw

#21 hemodiafiltration or haemodiafiltration:ti,ab,kw

#22 "peritoneal dialysis":ti,ab,kw

#23 ESKF or ESRF or ESRD or ESKD:ti,ab,kw

#24 CKF or CKD or CRF or CRD:ti,ab,kw

#25 "pre dialysis" or pre-dialysis:ti,ab,kw

#26 CAPD or CCPD or APD:ti,ab,kw

#27 MeSH descriptor: [Uremia] explode all trees

#28 uremia:ti,ab,kw

#29 MeSH descriptor: [Kidney Transplantation] explode all trees

#30 (kidney or renal) near (transplant* or graft* or allograft*):ti,ab,kw

#31 #1 or #2 or #3 or #4 or #5 or #6 or #7 or #8 or #9 or #10 or #11 or #12 or #13 or #14 or #15 or #16 or #17 or #18 or #19 or #20 or #21 or #22 or #23 or #24 or #25 or #26 or #27 or #28 or #29 or #30

#32 MeSH descriptor: [Gout Suppressants] explode all trees

#33 "gout suppressants":ti,ab,kw

#34 MeSH descriptor: [Urate Oxidase] explode all trees

#35 "uric acid" near lowering:ti,ab,kw

#36 allopurinol*:ti,ab,kw

#37 oxypurinol*:ti,ab,kw

#38 "xanthine oxidase inhibitor" or "xanthine oxidase inhibitors":ti,ab,kw

#39 benzbromarone*:ti,ab,kw

#40 probenecid*:ti,ab,kw

#41 sulfinpyrazone*:ti,ab,kw

#42 febuxostat*:ti,ab,kw

#43 uricase*:ti,ab,kw

#44 rasburicase*:ti,ab,kw

#45 peg-uricase or "peg uricase":ti,ab,kw

#46 pegloticase*:ti,ab,kw

#47 #32 or #33 or #34 or #35 or #36 or #37 or #38 or #39 or #40 or #41 or #42 or #43 or #44 or #45 or #46

#48 #31 and #47
