# Supplementary figures and images for: Effects of uric acid-lowering therapy in patients with chronic kidney disease: A meta-analysis
Source: PLoS One. 2017 Nov 2;12(11):e0187550. doi: 10.1371/journal.pone.0187550 (PMC5667873; doi:10.1371/journal.pone.0187550)

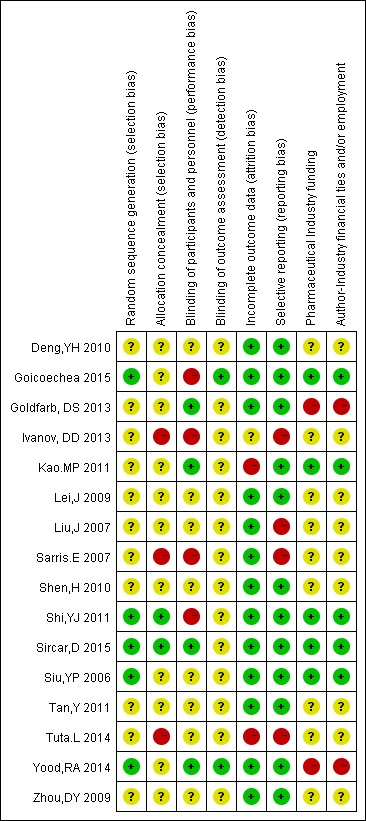

Supplement: S1 Fig — Only abstract was available in Sarris.E 2007, Ivanov, DD 2013 and Tuta, L 2014. (PNG) [file pone.0187550.s004.png]

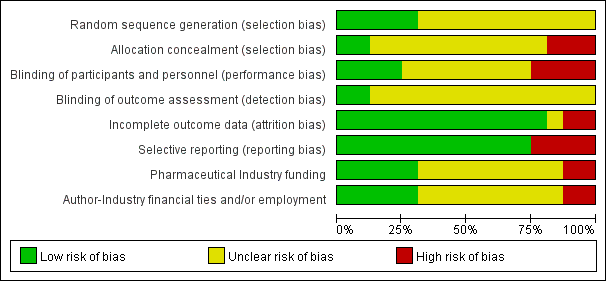

Supplement: S2 Fig — (PNG) [file pone.0187550.s005.png]
